# Supplementary material for: The rise in domestic shigellosis and the genomic characteristics of Shigella clones linked to men who have sex with men in Taiwan, 2015‒2022
Source: Microbiol Spectr. 2025 Feb 13;13(4):e02290-24. doi: 10.1128/spectrum.02290-24 (PMC11960102; doi:10.1128/spectrum.02290-24)
Supplement: Supplemental figures — Fig. S1 to S5. [file spectrum.02290-24-s0001.pdf]

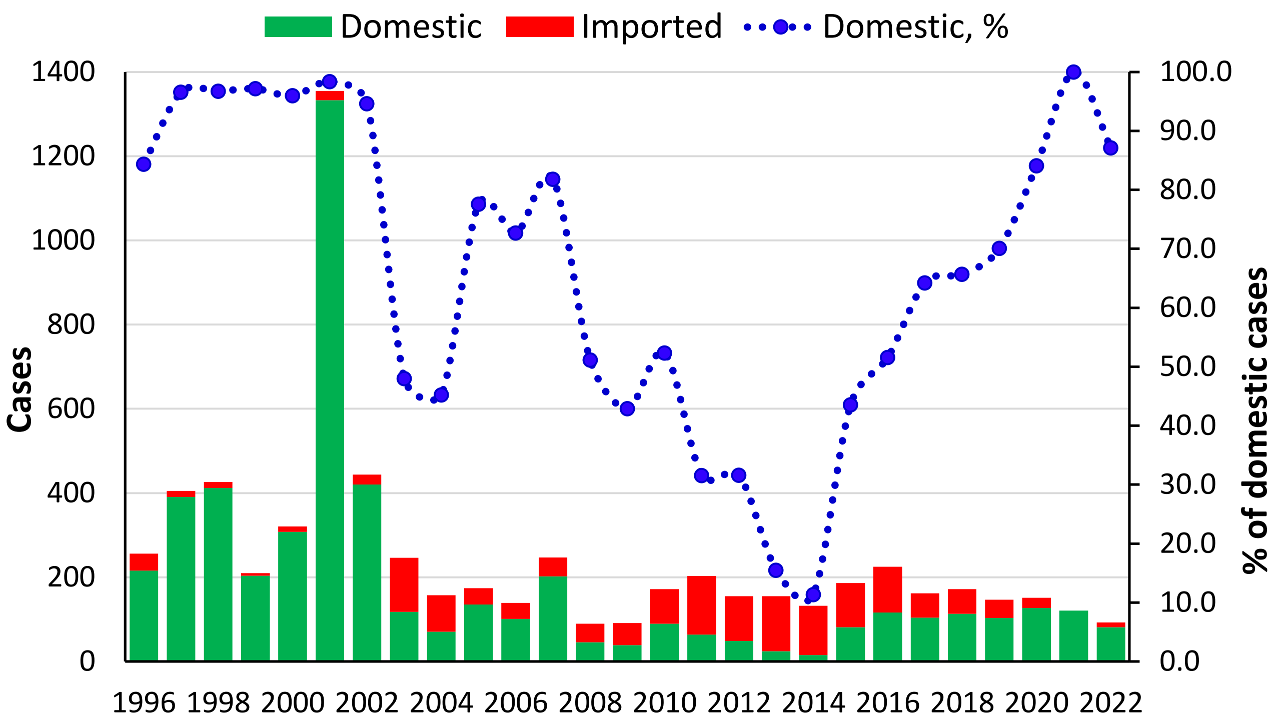

Figure S1. Epidemiological trend of shigellosis in Taiwan

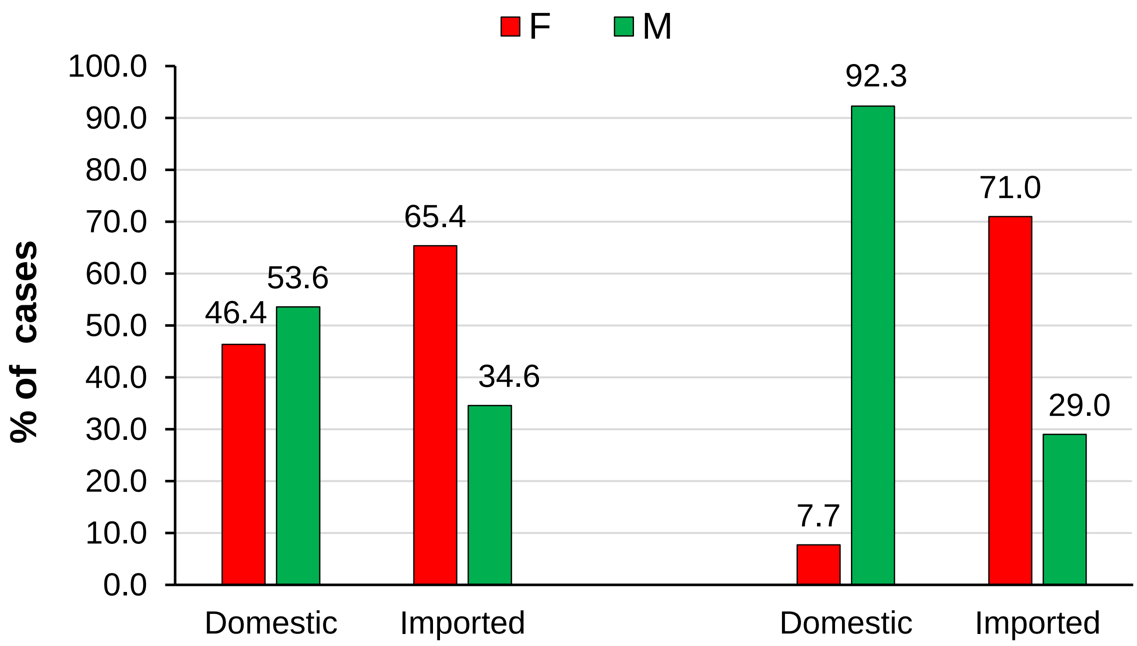

Figure S2. Sex-based distribution of shigellosis cases across the periods 2003–2014 and 2015–2022

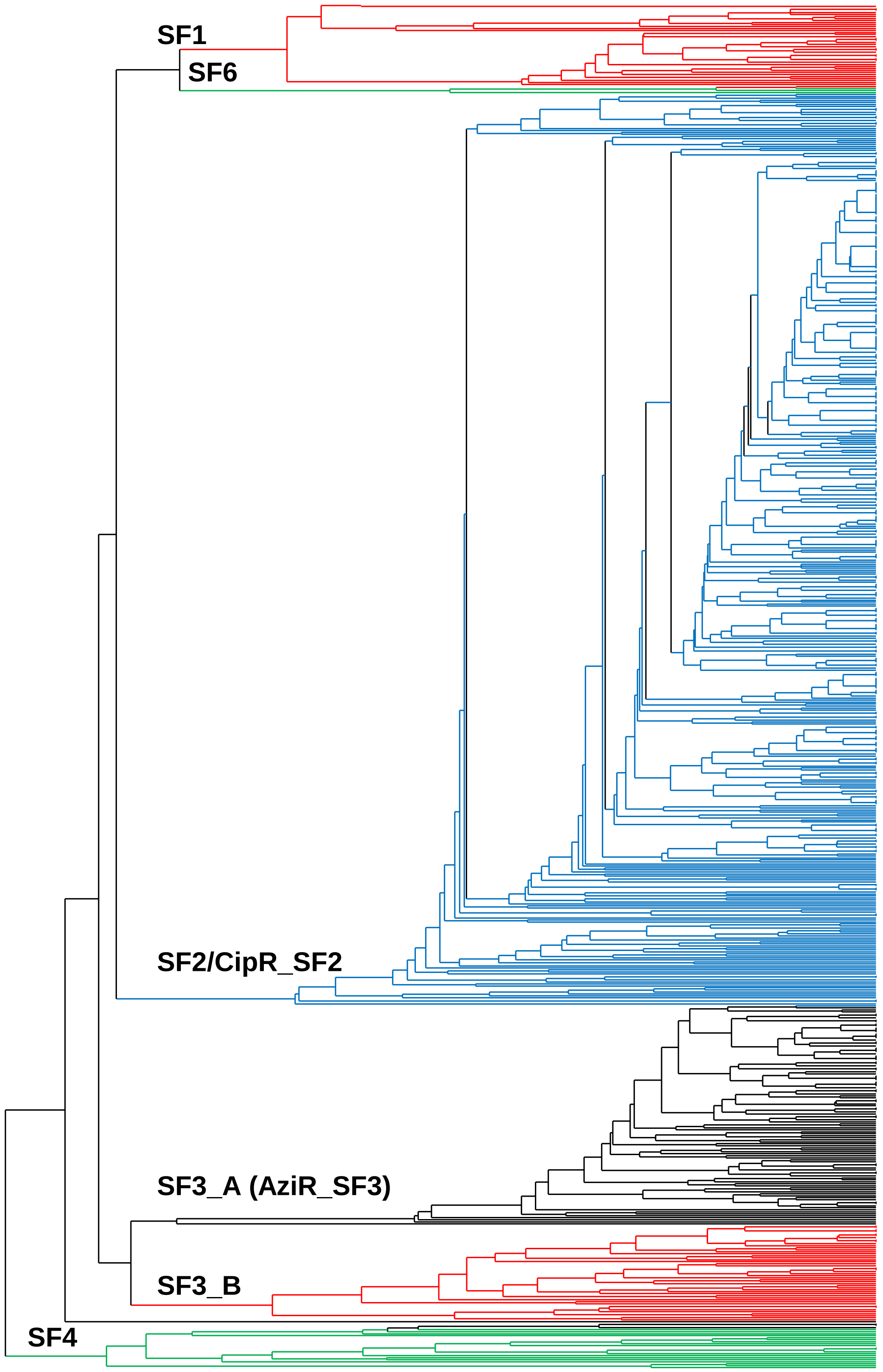

Figure S3. Phylogenetic tree constructed using PFGE patterns of 683 *Shigella flexneri* isolates. The tree was generated using the Dice coefficient and UPGMA algorithm, with a pattern optimization setting of 1.5% and a band match tolerance of 0.35%. The clusters SF1, SF2, SF3_A (AziR_SF3), SF3_B, SF4, and SF6 correspond to *S. flexneri* serotypes 1, 2, 3, 3, 4, and 6, respectively. The majority of SF2 isolates are identified as *S. flexneri* 2a, while SF3_A and SF3_B represent *S. flexneri* 3a.

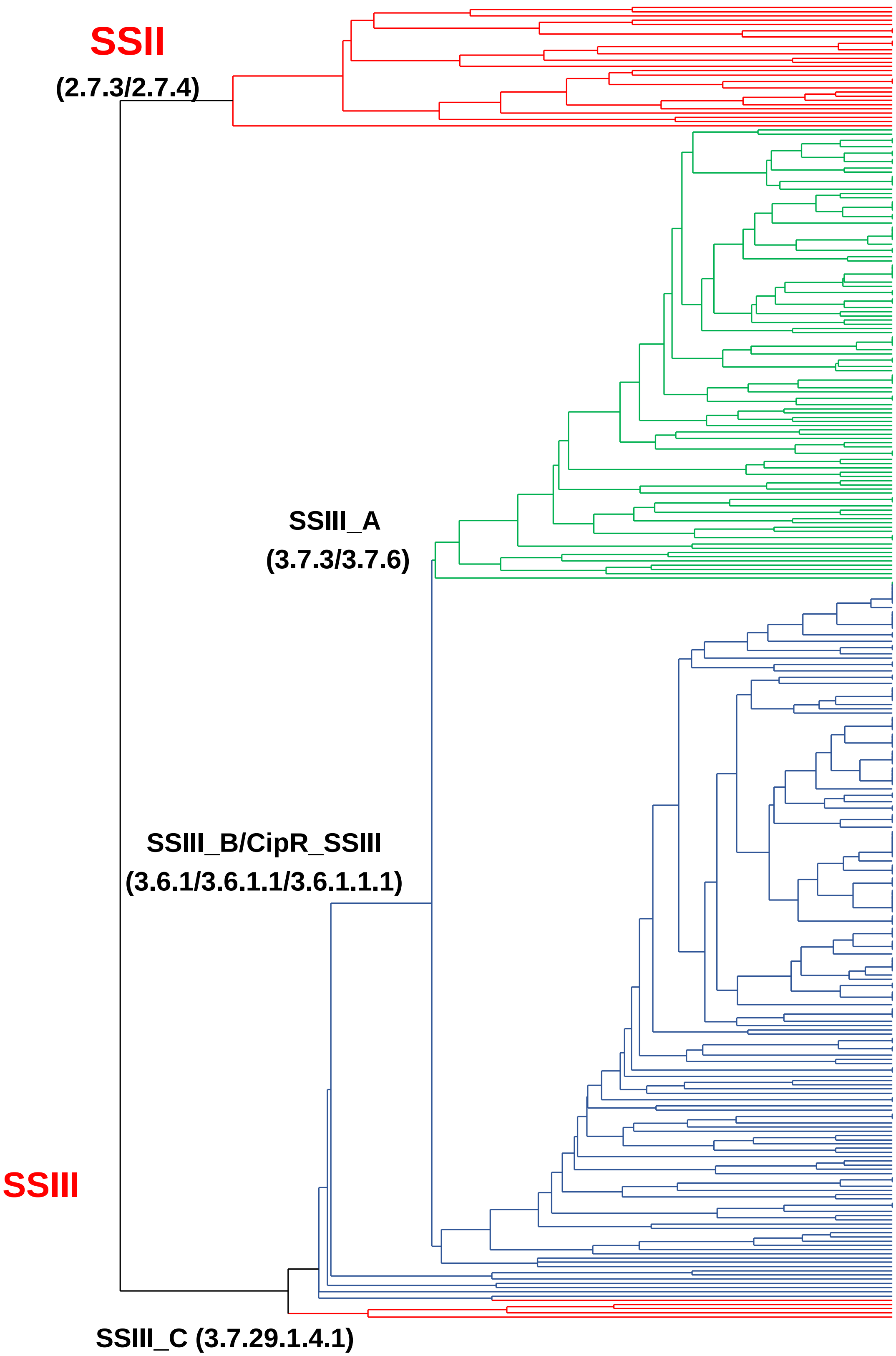

Figure S4. Phylogenetic tree constructed using PFGE patterns of 311 *Shigella sonnei* isolates. The tree was generated using the Dice coefficient and UPGMA algorithm with a pattern optimization setting of 1.5% and a band match tolerance of 0.35%. The clusters SSII and SSIII (subdivided into SSIII_A, SSIII_B/CipR_SSIII, and SSIII_C) correspond to *S. sonnei* genetic lineages II and III, respectively, as defined in the study by Holt et al. [1]. SSII isolates belong to Mykrobe genotypes 2.7.3 and 2.7.4. SSIII_A isolates are classified under genotypes 3.7.3 and 3.7.6, SSIII_B under genotypes 3.6.1, 3.6.1.1, and 3.6.1.1.1, and SSIII_C under genotype 3.7.29.1.4.1.

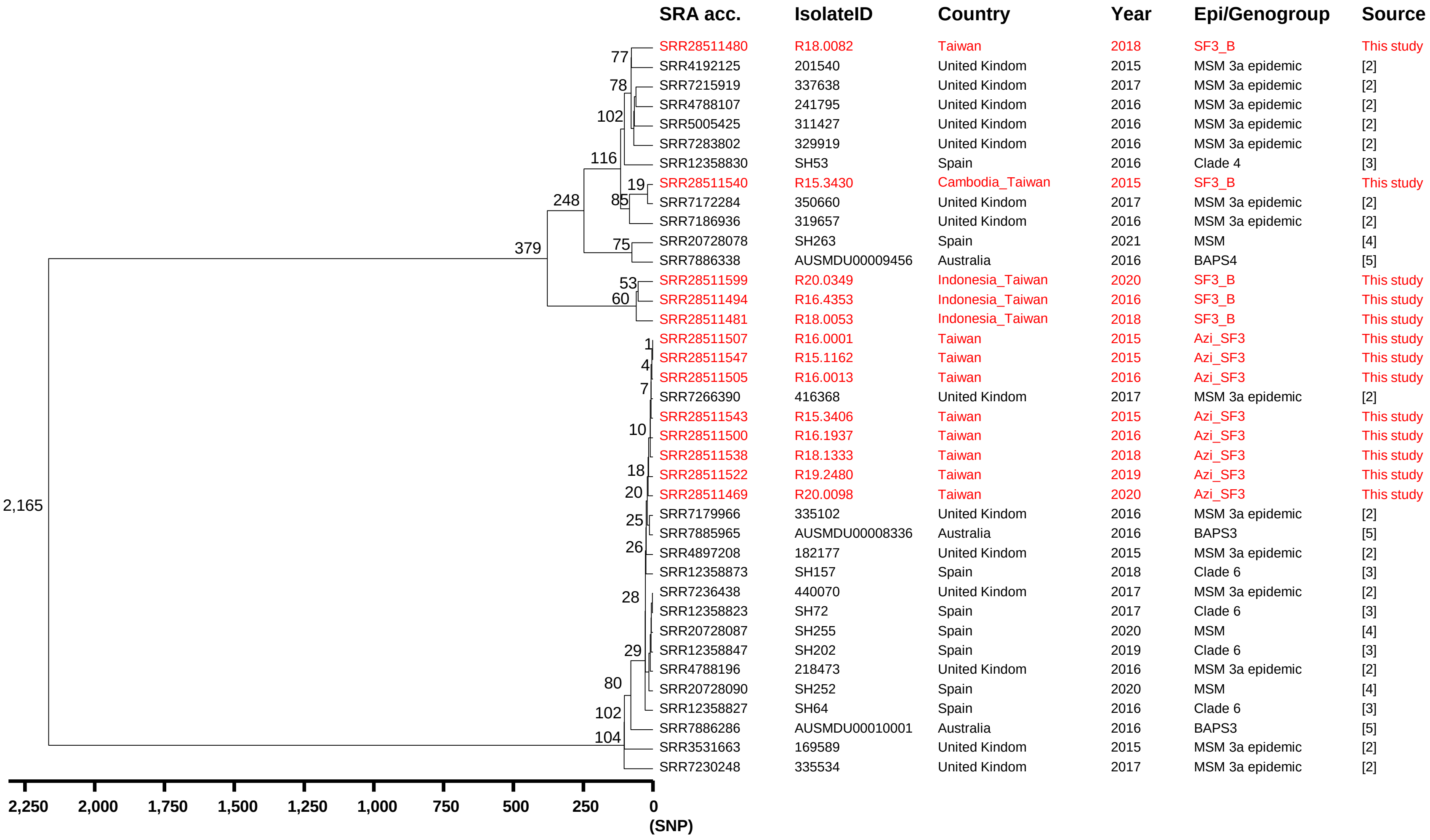

Figure S5. Phylogenetic tree of *Shigella flexneri* 3a isolates from Taiwan, compared with isolates from shigellosis outbreaks linked to MSM groups in Australia, Spain, and the United Kingdom. The tree was constructed using core genome SNP (cgSNP) profiles with the single-linkage algorithm, utilizing *S. flexneri* 2a strain 2457T as the reference for SNP calling.

References

[1] Holt KE, Baker S, Weill FX, et al. *Shigella sonnei* genome sequencing and phylogenetic analysis indicate recent global dissemination from Europe. Nat Genet 2012;44:1056-1059. doi:10.1038/ng.2369.

[2] Bardsley M, Jenkins C, Mitchell HD, et al. Persistent transmission of shigellosis in England is associated with a recently emerged multidrug-resistant strain of *Shigella sonnei*. J Clin Microbiol 2020;58:e01692-01619. doi:10.1128/JCM.01692-19.

[3] Moreno-Mingorance A, Espinal P, Rodriguez V, et al. Circulation of multi-drug-resistant *Shigella sonnei* and *Shigella flexneri* among men who have sex with men in Barcelona, Spain, 2015-2019. Int J Antimicrob Agents 2021;58:106378. doi:10.1016/j.ijantimicag.2021.106378.

[4] Moreno-Mingorance A, Mir-Cros A, Goterris L, et al. Increasing trend of antimicrobial resistance in *Shigella* associated with MSM transmission in Barcelona, 2020-21: outbreak of XRD *Shigella sonnei* and dissemination of ESBL-producing *Shigella flexneri*. J Antimicrob Chemother 2023;78:975-982. doi:10.1093/jac/dkad031.

[5] Ingle DJ, Easton M, Valcanis M, et al. Co-circulation of multidrug-resistant *Shigella* among men who have sex with men in Australia. Clin Infect Dis 2019;69:1535-1544. doi:10.1093/cid/ciz005.
